# Evaluation of Guided Bone Regeneration in Critical Defects Using Bovine and Porcine Collagen Membranes: Histomorphometric and Immunohistochemical Analyses

**DOI:** 10.1155/2021/8828194

**Published:** 2021-03-29

**Authors:** Guilherme A. D. Ramires, Julia Taino Helena, Júlio C. S. De Oliveira, Leonardo Perez Faverani, Ana Paula F. Bassi

**Affiliations:** ^1^Department of Diagnosis and Surgery, Araçatuba Dental School UNESP, Araçatuba 16015-050, Brazil; ^2^Department of Dentistry, Ceuma University, São Luis 65075-120, Brazil

## Abstract

Guided bone regeneration (GBR) is a technique used to facilitate bone regeneration, which uses a biocompatible membrane acting as a physical barrier to prevent the adjacent connective tissue from invading the bone defect. The aim of this study was to evaluate and compare the effectiveness of bovine and porcine collagenous membranes as barriers to connective tissue invasion during the repair of critical bone defects in rat calvaria, using histological, histometric, and immunohistochemical analyses. For this study, 72 rats were divided into three groups: clot group (CG), bovine collagen group (BCG), and porcine collagen group (PCG). Analyses were performed on days 7, 15, 30, and 60. The histological results showed that the PCG exhibited bone neoformation starting from day 7, and after 30 days of repair, the surgical defect was completely filled in some animals. For the BCG, there was little bone neoformation activity in the initial periods, and from day 30 onwards, there was an increase in bone neoformation, with a greater increase on day 60. The data obtained in the histometric analysis reveal that, on day 30, the neoformed bone area did not vary greatly between the PCG and the BCG, though both varied from the CG. By day 60, the PCG presented a greater area of neoformation than the BCG. These results were corroborated by the immunohistochemistry results. In view of the results obtained, it can be concluded that all membranes studied in this research promoted GBR.

## 1. Introduction

One of the great challenges for dentistry and medicine is the search for bone substitutes that meet the requirements for the physical-biological reconstruction of bone defects caused by traumatic physiological changes or pathological conditions [1, 2]. Thus, membranes appear to be a good option in guided bone regeneration (GBR) because they support osteopromotion, biocompatibility, noncytotoxicity, and mechanical stability, i.e., the ability to maintain space during the bone repair process [3–7].

The literature shows that certain tissues inside the body have the biological potential for regeneration during healing under adequate surrounding conditions [8, 9]. The main indications for the use of biological membranes in ROG processes are the correction of edentulous edges or residual defects [10], alveoli after extractions and dehiscence, and fenestrations after placement of mediate and immediate implants [11]. For bone neoformation to proceed, the following conditions must exist: there must be a source of osteogenic cells (viable bone adjacent to the defect); adequate vascularization must exist; the wound site must remain mechanically stable during healing (as micromovements may influence tissue formation); there must be an appropriate space between the membrane and the bone surface, preventing the collapse of the membrane in this critical space (thus, the space is filled by a blood clot in which the osteogenic cells will multiply); the membranes must have permeability properties that allow the diffusion of plasma and nutrients but not the passage of nonosteogenic cells; the membrane must be biocompatible; and the delicate vascular network must be protected during the clot organization [12].

According to Andrade-Acevedo [13], the problems most frequently associated with GBR and use of membranes are the total or partial collapse of the membrane, membrane exposure due to soft tissue dehiscence (local infection), and a poor technical skill. Options in the market include absorbable and nonabsorbable membranes. The first membranes used for GBR were nonresorbable. They required subsequent surgery to remove and were often associated with exposure problems and reduced clinical success [14]. Currently, the most researched and used membrane material in GBR procedures consists of a structure specifically formed by expanded polytetrafluoroethylene (e-PTFE) that cannot be chemically broken down under physiological conditions. Despite the high predictability of bone regeneration using e-PTFE membranes, the main disadvantage is that its exposure can cause bacterial contamination. The inflammatory reaction of the area may necessitate early removal of the membrane [15]. Several authors have reported a reduction in the amount of bone regenerated in these situations [15–17].

Absorbable membranes must undergo resorption and macromolecular degradation through hydrolysis and enzymatic degradation, which occurs in the presence of enzymes, such as acid phosphatase and collagenase. Bioreabsorption requires the total elimination of degradation products without local residual effects [18]. These membranes were created to eliminate a second surgical procedure. These biomaterials have been extensively investigated. A new generation of membranes consisting of polylactide-polyglycolide copolymer and polylactic acid has been studied [14].

Among the available membranes, collagen membranes meet most of the requirements: they are biocompatible and hemostatic, they promote chemotaxis for fibroblasts and osteoblasts, and being semipermeable, they allow the transfer of nutrients [19–24] and significant repair of intraosseous defects in the periodontium [25]. Furthermore, when associated with various types of bone grafts, collagen membranes can improve their effectiveness, increasing the ability to stimulate the repair of periodontal tissues [26–28]. Currently, alternatives are being sought to assist bone tissue repair and also to reduce the cost to the patient with high-performance and low-cost biomaterials [29]. The Lumina-coat® bovine collagen membrane—used for oral tissue regeneration—prevents soft tissue permeation into the bone defect, provides guidance for the proper formation of bone, soft tissue, and blood vessels, vascularizing it homogeneously during the healing process, leads to a good integration of the membrane with the adjacent tissue, and has a reduced cost [30, 31].

The aim of the present study was to evaluate, through histomorphometric and immunohistochemical analysis, the potential of bovine collagen and porcine collagen membranes in the process of guided bone regeneration to repair critical bone defects in rat calvaria.

## 2. Materials and Methods

The study was submitted to and approved by the Ethics Committee on Animal Experiments at the Araçatuba Dental School, Universidade Estadual Paulista (protocol number 01062-2017), and followed the ARRIVE Guidelines.

Seventy-two male 3- to 4-month-old rats (Rattus norvegicus albinus, Wistar) weighing approximately 200 to 300 g were randomly divided into 3 groups (24 rats per group). They were euthanized 7, 15, 30, or 60 days after surgery. The rats were kept in cages, 3 animals per cage, at the vivarium of the School of Dentistry, São Paulo State University (UNESP), Araçatuba. The rats were fed a balanced diet (NUVILAB, Curitiba, PR, Brazil) containing 1.4% calcium and 0.8% potassium and had free access to water.

### 2.1. Surgical Procedure

The surgical procedure was performed in the morning at the vivarium of the School of Dentistry, Sa˜o Paulo State University (UNESP), Araçatuba. The animals were subjected to preoperative fasting for 12 hours before being sedated by intramuscular administration of ketamine hydrochloride (50 mg/kg Francotar; Virbac do Brasil Ltda., Sa˜o Paulo, Brazil) combined with xylazine (5 mg/kg Rompun; Bayer S. A. Animal Health, Sa˜o Paulo, Brazil). Trichotomy was performed in the cranial calvaria region. Antisepsis procedures were performed with polyvinylpyrrolidone-iodine (PVPI, 10% Riodeine; Rioquimica, Sa˜o Jose do Rio Preto, Brazil) and topical PVPI (10% Riodeine; Rioquimica, Sa˜o Jose do Rio Preto, Brazil), and the rats were placed in a sterile field.

A V-shaped incision of approximately 1 cm on each side was made in the scalp in the anterior region of the calvarium, allowing for the reflection of a full-thickness flap in the posterior direction. An 8 mm diameter critical-sized defect was made with a 7 mm internal diameter trephine (3i Implant Innovations, Inc., Palm Beach Gardens, USA) housed in a low-speed handpiece with continuous irrigation with sterile saline. The defect was made in the central portion of the calvarium involving the sagittal suture to maintain the integrity of the dura mater. Depending on the proposed treatments, the defects were variously filled with blood clots without affixing the membrane (CG), and after affixing bovine collagen membrane (BCG), or porcine collagen membrane (PCG).

After the procedure, the soft tissues were carefully repositioned and sutured at different planes: resorbable suture thread (polylactic acid, Vicryl 4.0; Ethicon, Johnson Prod., Sa˜o José dos Campos, Brazil) was used at deep levels, and a monofilament thread (mononylon, Nylon 5.0; Ethicon, Johnson Prod.) with interrupted sutures was used at the most external plane.

In the immediate postoperative period, each animal received a single intramuscular dose of 0.1 mL penicillin G benzathine (Veterinary Pentabiotic for Small Animals; Fort Dodge Saude Animal Ltda., Campinas, SP, Brazil). The animals were euthanized at 7, 15, 30, and 60 days postoperatively by an overdose of anesthetic (sodium thiopental, 150 mg/kg). The calvaria was removed and fixed in 10% formaldehyde solution for 48 hours, washed in running water for 24 hours, decalcified in 20% EDTA for 7 weeks, dehydrated in alcoholic solutions, and diaphanized. The prepared calvaria was cut in the middle in the longitudinal direction to separate the bone defects. The pieces thus obtained were added individually to paraffin, and 6 *μ*m thick sections were obtained. The sections on slides were stained using hematoxylin and eosin.

### 2.2. Histomorphometric Analysis

All morphological analyses were performed using a binocular optical microscope with ×6.3, ×12.5, ×25, and ×40 lenses with an attached AxioCam ICc camera (Carl Zeiss, Oberkochen, Germany) to record images of the tissue sections.

The scanned images were saved in TIFF files. The histomorphometric analysis was performed using the images obtained with a 2.5*x*/0.07 objective. The panoramic images of the slides were assembled using Photoshop CS6 software, using the Photomerge tool. The area of bone tissue present throughout the panoramic image obtained from the slide was evaluated, excluding only the bone area of the stumps.

### 2.3. Immunohistochemical Analysis

Immunohistochemistry involved the detection of immunoperoxidase activity. Endogenous peroxidase activity was inhibited by hydrogen peroxide. Subsequently, the slides were processed for antigen recovery using phosphate-citrate buffer (pH 6.0), and endogenous biotin was blocked using nonfat dry milk. Primary antibodies against osteocalcin (Santa Cruz Biotechnology, Dallas, TX, USA) and osteopontin (Santa Cruz Biotechnology) were used. Antigenic recovery with citrate buffer at 60°C for 20 min was followed by blocking nonspecific reactions with skim milk and bovine albumin during antibody incubations. The polyclonal biotinylated secondary goat antibody produced in donkeys (Jackson ImmunoResearch Laboratories, West Grove, PA, USA) was used with an Avidin and Biotin Amplifier Kit (Vector Laboratories, Burlingame, CA, USA). Diaminobenzidine (Dako, Carpinteria, CA, USA) was used as the chromogen. For each antibody, the immunolabeling intensity of the relevant proteins was assessed semiquantitatively by assigning different scores, according to the number of cells immunolabeled in the bone repair process. The analysis was performed using an optical microscope (Leica DM4000B LED, Heerbrugg, Switzerland), using the following scores: null marking (0), mild marking (1), moderate marking (2), and intense marking (3). Immunostaining was represented by the average percentage of labeling for each protein evaluated. Thus, score 1 showed about 25% immunolabeling; score 2, 50%; and score 3, 75% of immunolabeling. Immunolabeling is known to be an indicator of the dynamics of bone tissue, and diaminobenzidine markings were considered positive, taking care to carry out negative controls (clot without membrane) to assess the specificity of the antibodies [32].

### 2.4. Statistical Analysis

The data obtained in the analyses were converted into absolute values of pixels, then to relative percentage values, in order to minimize the interference of the size difference between the images. To compare the average values obtained in the different groups and experimental periods, they were initially subjected to the statistical test of homoscedasticity, for later application of the most appropriate test (parametric or nonparametric) depending on the distribution of the data on the normality curve. Analyses were made using the statistical program for biological research, Sigma Plot 12.3 (Systat Software, Inc., San Jose, CA, USA), in which the level of significance was set at *p* < 0.05 for all tests.

## 3. Results

### 3.1. Histomorphometric Analysis

#### 3.1.1. CG

**Figure 1:**
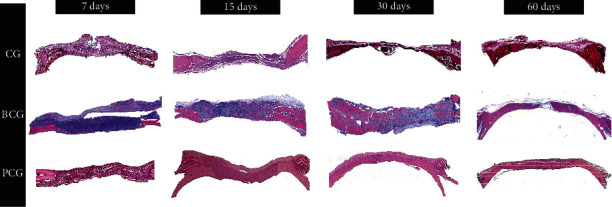
Photomicrographs of the histological sections at a lower magnification (2.5x) for the experimental groups (CG, BCG, and PCG) in all periods analyzed (7, 15, 30, and 60 days), in which we observed the bone repair capacity of each membrane.

In the clot group (CG), in the first 7 days, the images suggested hypervascularization with fibroblastic activity. On day 15, the edge of the bone defect moved towards the center of the wound, new bone forming only in the stumps of the surgical wound. Primary bone tissue was randomly distributed and rich in osteocytes and its surfaces were covered by osteoblasts. On day 30, a newly formed bone area closer to the stump of the defect was observed. The space between them was filled with loose, unmodified connective tissue. On day 60, the defect was filled with immature connective tissue and the presence of inflammatory infiltrate, with no sign of bone neoformation, confirming these to be critical defects (see Figure 1).

#### 3.1.2. BCG

**Figure 2:**
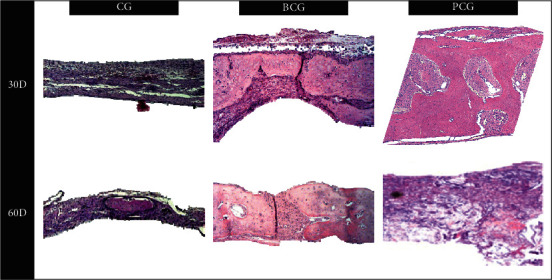
Photomicrographs of sections stained with HE, 30 and 60 postoperative days with a greater increase (CG, BCG, and PCG) (original 12.5x magnification).

In the bovine collagen group (BCG) on day 7, a well-organized granulation tissue was observed without clear signs of an inflammatory process, and a membrane was in place over the defect with cellular invasion. On day 15, the presence of granulation tissue interspersed with neoformed bone tissue; next to the stump, the center of the defect showed organized connective tissue, and a membrane was still present with islands of bone neoformation inside. On day 30, neoformed bone tissue was present in the center of the defect. On day 60, there was mature neoformed bone tissue interspersed with connective tissue, no remnant of the membrane, and blood vessels in the midst of the newly formed bone areas (see Figures 1 and 2).

#### 3.1.3. PCG

In the porcine collagen group (PCG), the presence of the porcine collagen membrane was noted in every defect filled probably by a highly vascularized tissue, without an inflammatory infiltrate on day 7. Tissues with less vascularization were also present, and areas of newly formed bone tissue from the stump were observed. There were also spots of osteoid tissue in the center of the defect on day 15. There was a large amount of neoformed bone tissue interspersed with fragments of the porcine collagen membrane on days 30 and 60, signs of complete closure of the defect in some specimens, and in others, the defect was almost closed, without the presence of membrane remnants (see Figures 1 and 2).

### 3.2. Immunohistochemical Analysis

Immunomarkings were evaluated in the central region of the critical defect using osteopontin (OP) to characterize the stages of bone neoformation and osteocalcin (OC) for bone mineralization. OC is a marker observed in osteoblasts in the final stage of mineralization, characterizing a more mature bone tissue, while OP is a marker positively observed in osteocytes; therefore, it is a marker of immature/young bone tissue.

#### 3.2.1. Osteocalcin-BCG

**Figure 3:**
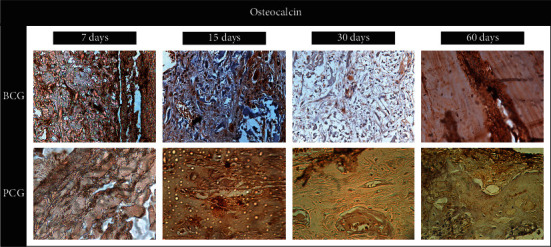
Photomicrographs at 40x magnification of histological sections from the center of the defect, sampled from the bovine collagen group (BCG) and porcine collagen group (PCG) at 7, 15, 30, and 60 days of repair, denoting areas of expression of the osteocalcin protein.

After 7 days, moderate positive marking for osteocalcin was observed close to the stumps and in the center of the defect (in the form of precipitates on the newly formed bone tissue), on osteocytes, and on connective tissue cells, thus indicating possible osteoblastic activity. After 15 days, an extracellular matrix positively marked for osteocalcin was observed, extending from the stump towards the center, with a moderate score (as per the immunohistochemical scoring system described earlier). After 30 days, bone trabeculae in formation were observed, positively marked for osteocalcin with a moderate score. After 60 days, moderate to intense immunostaining was noted, showing bone maturation and closure of the defect (see Figure 3 and Table 1).

#### 3.2.2. Osteocalcin-PCG

In PCG, immunostaining for osteocalcin with a moderate score for osteocalcin was observed in the extracellular matrix. After 15 days, immunostaining for this protein was observed with a moderate score in the extracellular matrix. After 30 and 60 days, immunostaining with a mild score was observed in the extracellular matrix (see Figure 3 and Table 1).

#### 3.2.3. Osteopontin-BCG

The extracellular matrix was positively immunostained for osteopontin, with a moderate score, after 7 and 60 days. Mild immunostaining was observed after 15 and 30 days.

#### 3.2.4. Osteopontin-PCG

After 7 days, it was possible to observe an extracellular matrix positively immunostained for osteopontin with a moderate score, showing the beginning of the bone formation process. After 15 days, moderate immunostaining for this protein was observed. After 30 and 60 days, mild immunostaining was observed for this protein; this is consistent with bone maturation, during which there is less marking for this protein.

### 3.3. Statistical Analysis

All tests were performed using the Sigma Plot 12.3 statistical program (Systat Software, Inc., San Jose, CA, USA). Initially, the data were submitted to the normality test (Shapiro–Wilk), which did not identify homogeneous data (*p* < 0.05); that is, the test failed. Thus, the Kruskal–Wallis test was applied, and all interactions showed statistically significant changes (*p* < 0.05). Additionally, for the precise identification of statistical changes, the Tukey posttest was applied, considering a significance level of 5%.

**Figure 4:**
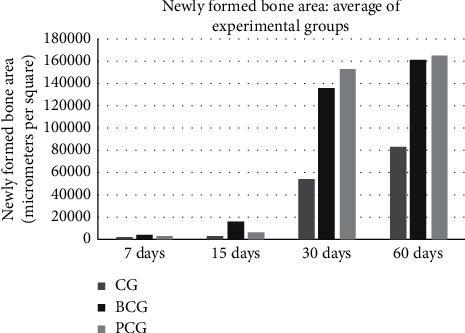
Newly formed bone area for experimental groups.

In the histometric evaluation of the chronological evolution of bone repair (intragroup analysis), the experimental group (BCG) showed a greater area of bone neoformation at 60 days compared to the other periods analyzed (7, 15, and 30 days) (*p* < 0.05). However, there was no statistical difference in bone formation between the 30-day period and the 60-day period (*p* = 0.745). Compared with the control groups, the results of the CG (negative control) and PCG (positive control) were similar between the periods of 7 and 15 days (*p* = 0.985), and between 30 and 60 days (*p* = 0.422), showing a difference only when the two initial periods were compared with the two final periods (*p* < 0.05) (see Figure 4).

The highest AON values were observed for the PCG, which were similar on days 30 and 60 (*p* = 0.721). There was also similarity between the BC and PC groups in the period between 30 (*p* = 0.762) and 60 days (*p* = 0.980) (see Table 2).

## 4. Discussion

The main objective of GBR is to obtain new bone with adequate volume and quality in the area of a critical defect, with high predictability and low risk of complications. Second, GBR is used to obtain a good result with fewer surgical interventions, low morbidity for the patient, and a reduced repair period. It is known that, in the last 20 years, there have been significant advances in the development of techniques and materials for GBR to occur in a predictable way [33, 34].

Critical-sized bone defects are those that are not able to regenerate spontaneously throughout the life of the animal, that is, without the aid of a biomaterial or a membrane for osteoconduction. Therefore, the use of biomaterials and/or autogenous grafts assists bone repair, so these repairs will be significantly superior when compared to a repair using only the clot [35]. The bone defect created in this study, measuring 8 mm in external diameter, is considered critical, according to several studies [35–37], and corroborated by the results presented here, since it was possible to verify both by histological analysis and by histometric analysis that the defect could not be repaired without intervention. Thus, the use of a negative control group (clot without membrane) aims to establish how repair occurs in a standard way; that is, that bone formation is restricted to the margins of defects and the center is filled with fibrous connective tissue.

When we add a mechanical barrier, that is, a membrane, it is expected that there will be an increase in the amount of newly formed bone and therefore a greater area of tissue regeneration, with greater bone neoformation when compared to the control [9]. Thus, we wanted to evaluate whether the GBR process occurs differently between different membranes and whether the quantity and/or quality of newly formed bone differs.

At present, we have a wide variety of membranes for GBR that are available in the market. The choice of the membrane must be based on clinical needs and must meet basic criteria, such as biocompatibility, cell occlusion, tissue integration, space formation and maintenance, ease of handling, and limited susceptibility to complications [38–41].

Collagen membranes are obtained from different animal tissues (tendon, skin, and intestine), bovine or porcine [38, 42]. Although collagen has numerous advantages, such as low immunogenicity, attraction of gingival fibroblasts, and biocompatibility [41, 42], the rate of degradation is high and the membrane may not be maintained long enough for adequate tissue repair [38]. The predictability of the collagen membrane depends not only on its composition, but also on the chemical and physical processes applied to this collagen to eliminate impurities, stabilize the fibers, and thus improve their mechanical properties and reduce the speed of collagen degradation [38, 41, 42].

With the results obtained in this research, it was possible to observe that the PCG membrane (Bio-Gide®) maintained a high level of performance, as described in the literature [43–46]. The membrane used in the PCG has a composition based on pure collagen types I and III of porcine origin, without crosslinking or chemical additives, which are refined to remove antigens [47]. The period of degradation described in the literature varies from 8 weeks [48] to 4–6 months [47, 49, 50]. In this study, the presence of the membrane was observed in the four periods evaluated by means of morphological analysis; the presence of giant cells was not observed, and it was not possible to observe an acute inflammatory reaction. Its resorption occurred in the form of lumps, suggesting partial resorption (approximately 8 weeks) and penetration of bone tissue, probably due to its high permeability, which allows blood to penetrate. After 30 and 60 days, more defects were closed, and the bone volume obtained after 30 days was maintained until the end of the repair process on day 60 [43, 49].

The membrane used in the BCG experimental group (Lumina-coat®) is composed of type I collagen extracted from the decalcified bovine bone cortex. The BC group presented fibrocellular connective tissue, and only fragments of the membrane were detected in the period of 30 days and, even less frequently, at 60 days. These results corroborate previous studies by Costa et al., Accorsi-Mendonca et al., and Bernabé et al., who observed that, at 30 days, there were only fragments of the membrane [35] or complete absence of it [36, 51].

In the PCG, bone neoformation was more progressive. A slower resorption process was observed through microscopic analysis compared to BCG. Multinucleated giant cells were observed on the outer surface of the membrane in the BCG group within 30 days, which suggests reabsorption of this biomaterial, as previously reported [35]. This was confirmed in the 60-day period, when a little remnant of the membrane was observed.

Comparing the histometry, the PC group presents a larger area of bone neoformation than does the BC group, which may be directly related to the nature of degradation of this membrane: it is possible to observe its presence in the later period, plus the presence of giant cells. Although a faster degradation of the membrane occurred in the PCG, the membrane expedited bone repair: the total volume of bone formed after 30 days was maintained until the end of the repair. Bovine collagen membrane, although its remnant was present after 60 days, functioned slightly less as a barrier than did the PCG, consequently allowing the growth of connective tissue in the center of the defect and inhibited bone neoformation; thus, there was no complete bone neoformation that would allow the closure of the defect.

The immunohistochemical evaluation showed different biological behaviors between the BC and PC groups. The PC group presented some important details, demonstrating better-sequenced events: osteopontin was more immunostained after 7 and 15 days, indicating that they have a greater presence of osteoid tissues, and after 30 and 60 days, osteopontin was only lightly marked. It is at this stage that osteocalcin increased its activity and presented itself as an intense immunomarker since mature bone tissue was already present. In the BC group, the protein, osteopontin, had mild immunostaining after 7 days and moderate after 15 days, maintaining this state until 60 days. This may be due to membrane hydrolysis and tissue disorganization delaying bone maturation in the region. As for osteocalcin marking for the BC group, it remained moderate in the initial periods and became intense on day 60, corroborating the histometric findings.

One of the main hindrances to this study was the scarcity of prior work using the bovine collagen membrane of this experiment, probably due to its recent commercialization. The results of this research demonstrate that this membrane has the potential to assist the GBR process, showing no statistical difference in bone neoformation in relation to PCG, which is a membrane already established with studies in the literature reporting its effectiveness.

## 5. Conclusions

Within the methodology employed, it was possible to conclude that the membranes investigated in this study promoted ROG in guided bone regeneration of critical defects in rat calvaria.

## Figures and Tables

**Table 1:** Representative scores related to the immunostaining of OC and OP proteins for BCG and PCG, with scores classified as null (0), mild (+), moderate (++), and intense (+++).

Groups	Osteocalcin	Osteopontin
BCG 7 days	++	++
BCG 15 days	++	+
BCG 30 days	++	+
BCG 60 days	+++	++
PCG 7 days	++	++
PCG 15 days	++	++
PCG 30 days	+++	+
PCG 60 days	+++	+

**Table 2:** Group vs. time comparison.

Group × time results	
7 days	*p* > 0.05 (similar)
15 days	*p* > 0.05 (similar)
30 days	(BCG = PCG (*p* = 0.762)) > CG (*p* < 0.05)
60 days	(BCG = PCG (*p* = 0.980)) > CG (*p* < 0.05)

## Data Availability

The data used to support the findings of this study are included within the article.
